# Swertiamarin ameliorates diet-induced obesity by promoting adipose tissue browning and oxidative metabolism in preexisting obese mice: Swertiamarin reverses obesity by enhancing energy expenditure

**DOI:** 10.3724/abbs.2022154

**Published:** 2022-10-26

**Authors:** Yuqin Zhu, Haoran Li, Mengchen Ma, Dandan Li, Ouyang Zhang, Suili Cai, Yajiao Wang, Dandan Chen, Shengnan Jin, Chunming Ding, Liang Xu

**Affiliations:** 1 School of Laboratory Medicine and Life Sciences Wenzhou Medical University Wenzhou 325035 China; 2 Key Laboratory of Laboratory Medicine Ministry of Education Wenzhou Medical University Wenzhou 325035 China

**Keywords:** obesity, swertiamarin, fat browning, energy expenditure, fatty acid oxidation

## Abstract

Obesity is a risk factor for many metabolic diseases. Efficient therapeutic strategies are urgently needed. Swertiamarin (STM) prevents obesity and the associated insulin resistance and inflammation. However, the therapeutic effects of STM on preexisting obesity remain unclear. Therefore, in this study we aim to investigate the effects of STM on energy expenditure and fat browning in mice with preexisting obesity. C57BL/6J mice are fed with a high-fat diet (HFD) for 8 weeks to induce obesity and then gavaged (or not) with STM for 10 weeks. The whole-body energy metabolism of mice is examined by indirect calorimetry. The results show that after 10 weeks of treatment, STM markedly prevents HFD-induced weight gain, chronic inflammation, insulin resistance, and hepatic steatosis. STM promotes oxygen consumption and energy expenditure. The level of uncoupling protein 1 is enhanced in the brown and white adipose tissues of STM-treated mice. STM increases the phosphorylation of AMP-activated protein kinase and the expressions of genes involved in fat oxidation, reducing fat deposition in skeletal muscles. Meanwhile, STM does not affect the intestinal microbiotic composition. Overall, STM supplementation may serve as a potential therapy for obesity.

## Introduction

Obesity is a risk factor for many metabolic diseases [1]. The global prevalence of obesity has been on the steady rise for the last few decades [ 1, 2] . Obesity is mostly caused by an imbalance between food intake and energy expenditure, which can be rectified by caloric restriction and/or increasing energy expenditure [3]. Restricting food intake to reduce body weight is difficult to maintain over the long term for many people. Thus, increasing energy expenditure is an attractive alternative for combating obesity [ 4, 5] .

Energy expenditure is mainly carried out to produce heat in brown adipose tissue (BAT) and skeletal muscle [6]. The adaptive thermogenesis of BAT is mediated by mitochondria with a high level of uncoupling protein 1 (UCP1) to convert energy stored in fat to heat, resulting in fat reduction [7]. Brown-like adipocytes (also known as beige adipocytes) in white adipose tissue (WAT) can also contribute to thermogenesis and energy expenditure via upregulation of UCP1 to protect against obesity [ 8, 9] . Skeletal muscle is also thermogenic by promoting fatty acid oxidation, which is mediated by AMP-activated protein kinase (AMPK) sensing low ATP/ADP ratios [ 10, 11] . Phosphorylation of Thr172 of catalytic subunit α activates AMPK, which then phosphorylates and inhibits downstream acetyl-CoA carboxylase (ACC) to promote lipid oxidation and decrease lipid deposition in peripheral tissues [12]. Therefore, adipose tissue browning and/or activation of AMPK-modulated catabolism may provide strategies for treating obesity by increasing energy expenditure [ 13, 14] .

Swertiamarin (STM) is a secoiridoid glycoside initially obtained from *Enicostemma littorale* Blume ( *E*. *littorale*). STM was found to be multifunctional, including mitigating diabetes [15], alleviating nonalcoholic steatosis (NAFLD) [16], and preventing cancer [17]. Wang *et al*. [18] found that STM attenuated lipid storage by activating 3-ketoacyl-coA thiolase. STM enhances hepatocyte AMPK phosphorylation, thereby attenuating oleic acid-induced adipogenesis and hepatic steatosis [19]. We previously showed that in a preventive model where STM was administered to mice concurrently fed with a high-fat diet (HFD), STM mitigated insulin resistance and inflammation [20]. However, the underlying mechanism remains unclear. More importantly, it is not known whether STM is effective in mice that are already obese.

In this study, we explored the effects of STM on energy metabolism to reduce obesity-associated insulin resistance and inflammation in mice with preexisting diet-induced obesity (DIO).

## Materials and Methods

### Animal study

Male C57BL/6 mice (8 weeks of age; Shanghai Laboratory Animal Center, Shanghai, China) were fed with normal chow (NC, 10% of calories from fat) or a HFD (60% of calories from fat) (D12492; Research Diets, New Brunswick, USA) for 8 weeks. Then, NC mice ( *n*=6) continued to consume NC, but HFD mice were divided into two body weight-matched groups: HFD ( *n*=9) and HFD+STM ( *n*=9). Both NC and HFD mice were given normal saline (via gavage) daily; HFD+STM mice received normal saline with 0.1% (w/w) STM (#90957, purity 98.4%; Sigma-Aldrich, St Louis, USA) for the next 10 weeks. Animal welfare and the experimental procedures adhered to the ethical provisions on the Care and Use of Experimental Animals of Wenzhou Medical University and were approved and authorized by the Animal Experimental Committee of the University (No. wydw2021-0111) on January 11, 2021.

### Indirect calorimetry

The oxygen consumption (VO _2_), carbon dioxide production (VCO _2_), and respiratory exchange ratio (RER) were determined after 3 weeks of STM treatment using a Comprehensive Lab Animal Monitoring System (CLAMS)-Oxymax (Columbus Instruments, Columbus, USA). The energy expenditure was calculated using the formula VO _2_×[3.815+(1.232×RER)] and analysed using ANCOVA with body weight as a covariate via the MMPC.org ANCOVA data analysis tool, as previously described [ 21, 22] .

### Body composition and biochemical analysis

Body composition was examined using Echo magnetic resonance imaging (Echo MRI, Houston, USA) prior to sacrifice. The levels of fasting plasma insulin, glucose, and triglycerides (TGs) were determined using commercial kits (Jiancheng, Nanjing, China). The homeostasis model assessment for insulin resistance (HOMA-IR) was calculated as fasting insulin (μU/L)×fasting glucose (nM)/22.5.

### Lipid isolation and identification

Lipids were extracted from the liver and the quadriceps femoris muscle (qM) via homogenization with isopropanol [23]. The levels of TG and nonesterified fatty acids (NEFAs) were examined using colorimetric assay kits (Jiancheng, Nanjing, China) and normalized to the tissue weight.

### Histological analysis

Fresh BAT, epididymal WAT, liver, and qM tissues were fixed in 10% (v/v) formalin, embedded in paraffin, and sectioned. The sections were stained with hematoxylin and eosin (H&E) or immunohistochemically using anti-F4/80 antibody (#70076; Cell Signaling Technology, Danvers, USA) and anti-UCP1 antibody (sc-518024, Santa Cruz Biotechnology, Santa Cruz, USA) [24]. In brief, the sections were heated (65°C, 2 h), dewaxed, rehydrated, and boiled (2 min) in sodium citrate (10 mM, pH6.0) for antigen retrieval. The slides were then treated with 3% H _2_O _2_ for 30 min to eliminate endogenous peroxidase activity and blocked with 5% BSA for 1 h. The slides were incubated with primary antibody at 4 °C overnight, followed by incubation with HRP-conjugated goat anti-rabbit secondary antibody (A0208; Beyotime, Shanghai, China) for 1 h at room temperature. Color development was performed by incubation with DAB reagent (P0203; Beyotime), and the slides were counterstained with hematoxylin according to manufacturer’s protocols.

### Insulin tolerance and glucose tolerance tests

Prior to the insulin tolerance test (ITT), mice were fasted for 4 h and injected intraperitoneally with 1 U/kg body weight human insulin (Sigma-Aldrich). Blood glucose levels were determined using a glucose meter at 0, 30, 60, 90, and 120 min. Glucose tolerance test (GTT) was performed after overnight fasting. The mice were then intraperitoneally injected with 2 g/kg glucose (Beyotime). Blood glucose levels were recorded before and at 30, 60, 90, and 120 min after injection.

### Real-time polymerase chain reaction

Real-time polymerase chain reaction (PCR) was used to determine mRNA expression. Briefly, total RNA was isolated from frozen tissues using TRIzol ^TM^ reagent (Invitrogen, Carlsbad, USA), and the concentration was determined with the NanoDrop-5000 spectrophotometer (Thermo Scientific, Waltham, USA). cDNA was synthesized using a high-capacity cDNA reverse transcription kit (Applied Biosystems, Foster City, USA) and mRNA expression levels were determined by RT-PCR using SYBR Green as described previously [20]. The primers used for real-time PCR are listed in Table 1.

**Table:** **Table 1** Mice primer sequences

Gene	Forward primer (5′→3′)	Reverse primer (5′→3′)
Acox1	TTATGCGCAGACAGAGATGG	AGGCATGTAACCCGTAGCAC
Ccr2	ATTCTCCACACCCTGTTTCG	GATTCCTGGAAGGTGGTCAA
Cd11c	AAAATCTCCAACCCATGCTG	CACCACCAGGGTCTTCAAGT
Cidea	ATCACAACTGGCCTGGTTACG	TACTACCCGGTGTCCATTTCT
Dio2	CAGTGTGGTGCACGTCTCCAATC	TGAACCAAAGTTGACCACCAG
Elovl3	TCCGCGTTCTCATGTAGGTCT	GGACCTGATGCAACCCTATGA
F4/80	CTTTGGCTATGGGCTTCCAGTC	GCAAGGAGGACAGAGTTTATCGTG
Fas	AGAGACGTGTCACTCCTGGACTT	GCTGCGGAAACTTCAGAAAAT
Mcp1	AGGTCCCTGTCATGCTTCTGG	CTGCTGCTGGTGATCCTCTTG
Pgc-1α	ATGTGTCGCCTTCTTGCTCT	ATCTACTGCCTGGGGACCTT
Pparα	GAGGGTTGAGCTCAGTCA GG	GGTCACCTACGAGTGGCATT
Scd1	CATCATTCTCATGGTCCTGCT	CCCAGTCGTACACGTCATTTT
Srebp-1c	GGAGCCATGGATTGCACATT	GGCCCGGGAAGTCACTGT
Tnf-α	AAGCCTGTAGCCCACGTCGTA	GGCACCACTAGTTGGTTGTCTTTG
Ucp1	ACTGCCACACCTCCAGTCATT	CTTTGCCTCACTCAGGATTGG
β-actin	AGGCCCAGAGCAAGAGAGGTA	GGGGTGTTGAAGGTCTCAAACA
18S	AGG CCC AGA GCA AGA GAG GTA	GGG GTG TTG AAG GTC TCA AAC A

### Western blot analysis

The protein expression was detected by western blot analysis. The frozen tissues were homogenized in RIPA lysis buffer (Millipore, Billerica, USA) supplemented with protease and phosphatase inhibitors (Sigma-Aldrich). The total protein concentration was determined using a BCA Protein Assay Kit (Pierce, Bonn, Germany). The lysates were blotted overnight at 4°C using primary antibodies ( Table 2) and then incubated with appropriate secondary antibodies (Cell Signaling Technology). The protein bands were visualized using chemiluminescence kit (Bio-Rad Laboratories Inc., Hercules, USA) and imaged using a gel imaging system (ImageLab, ver. 3.0; Bio-Rad).

**Table:** **Table 2** Antibodies used in immunoblotting

Antibody	Company
anti-phospho-AMPKα	Cell Signaling (#2535)
anti-AMPKα	Cell Signaling (#2532)
anti-phospho-p38 MAPK	Cell Signaling (#9211)
anti-p38 MAPK	Cell Signaling (#9212)
anti-phospho-NF-κB	Cell Signaling (#3033)
anti-NF-κB	Cell Signaling (#3034)
anti-phospho-Akt	Cell Signaling (#9271)
anti-Akt	Cell Signaling (#9272)
anti-UCP1	Abcam (ab10983)
anti-α-Tubulin	Cell Signaling (#2144)

### Gut microbiome profiling

Total genomic DNA was extracted from cecal contents using a QIAamp DNA Stool Mini Kit (Qiagen, Valencia, USA). The V3–V4 regions of 16S rRNA genes were amplified using the primer sets described previously [25]. Raw FASTQ files were quality-filtered by Trimmomatic and FLASH. UPARSE was employed to cluster the OTUs at 97% similarity [26]. The raw data were analysed using QIIME scripts, and microbial classification was performed with the aid of the GreenGenes reference database (gg_otus-13_8) using QIIME tools [27].

### Statistical analysis

Data are expressed as the mean±SEM. Differences between the two groups were assessed using the two-tailed, unpaired Student’s *t*-test or one-way ANOVA followed by post hoc comparisons employing the Tukey correction. GraphPad Prism ver. 8.3.0 was employed for all analyses. A *P* value<0.05 was considered statistically significant.

## Results

### STM attenuates body weight gain and adipocyte hypertrophy by promoting energy expenditure

**Figure 1:**
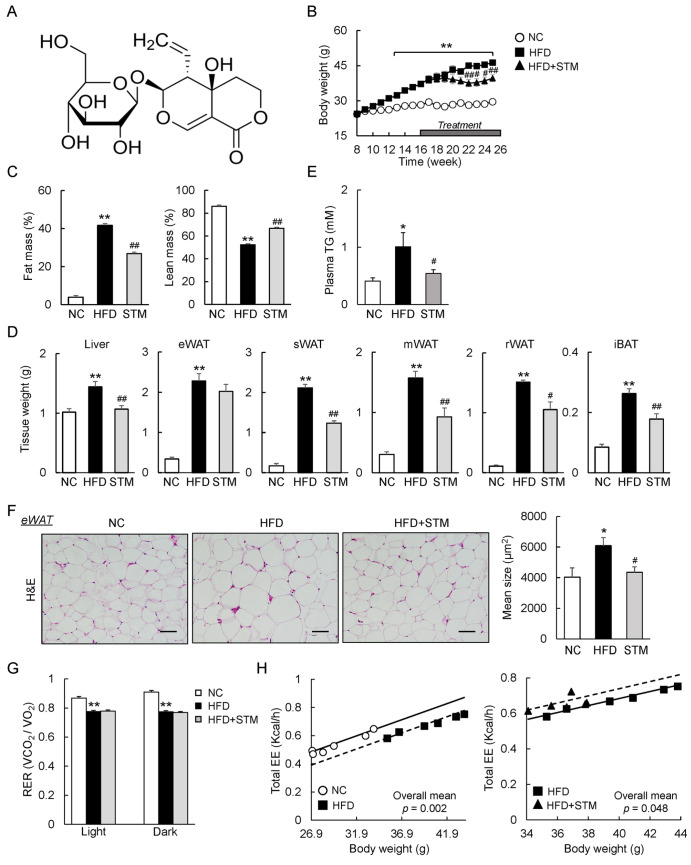
Figure 1 STM supplementation attenuates body weight gain and enhances energy expenditure (A) Chemical structure of swertiamarin. (B) Body weights. (C) Fat and lean masses. (D) Tissue weights. (E) Plasma TG levels. (F) eWAT sections stained with H&E (scale bar: 100 μm) and the mean eWAT adipocyte size. (G) Respiratory exchange ratios (VO 2/VCO 2, RERs). (H) Linear regression analysis of energy expenditure (EE) versus body weight by ANCOVA. Linear regression was plotted using https://www.mmpc.org/shared/regression.aspx. Data are presented as the mean±SEM. Significance was determined by one-way ANOVA. * P<0.05, ** P<0.01 vs NC; # P<0.05, ## P<0.01 vs HFD.

To evaluate the effect of STM ( Figure 1A) on body weight, obese mice were treated with 0.1% (w/w) STM for 10 weeks. STM significantly prevented further body weight gain ( Figure 1B). MRI showed that STM markedly reduced the fat mass and increased the lean mass of mice fed with the HFD ( Figure 1C). In addition, the liver weights of interscapular BAT and subcutaneous, mesenteric, and retroperitoneal WAT were decreased ( Figure 1D). STM reduced the plasma levels of TG ( Figure 1E) and adipocyte hypertrophy ( Figure 1F). Thus, the weight loss caused by STM was largely attributable to reductions in fat deposition and hypertrophy.

To examine whole-body energy homeostasis, we placed the mice in indirect calorimetry cages after 3 weeks of treatment before manifest alterations in the body weight of HFD+STM mice were observed. The metabolic cage data revealed that HFD mice exhibited lower RER (VO _2_/VCO _2_) than NC mice but similar RER to HFD+STM mice ( Figure 1G). Moreover, analysis of the energy expenditure data using ANCOVA with body weight as the covariant demonstrated that HFD reduced the total energy expenditure compared with NC ( *P*=0.002, Figure 1H), while STM treatment notably increased the total energy expenditure in HFD-fed mice ( *P*=0.048, Figure 1H). Unsurprisingly, the total energy expenditure was not significantly affected by 0.01% STM (w/w) (data not shown). Thus, the STM reversal of obesity was principally attributable to increased energy expenditure.

### STM improves BAT function and white adipocyte browning in obese mice

**Figure 2:**
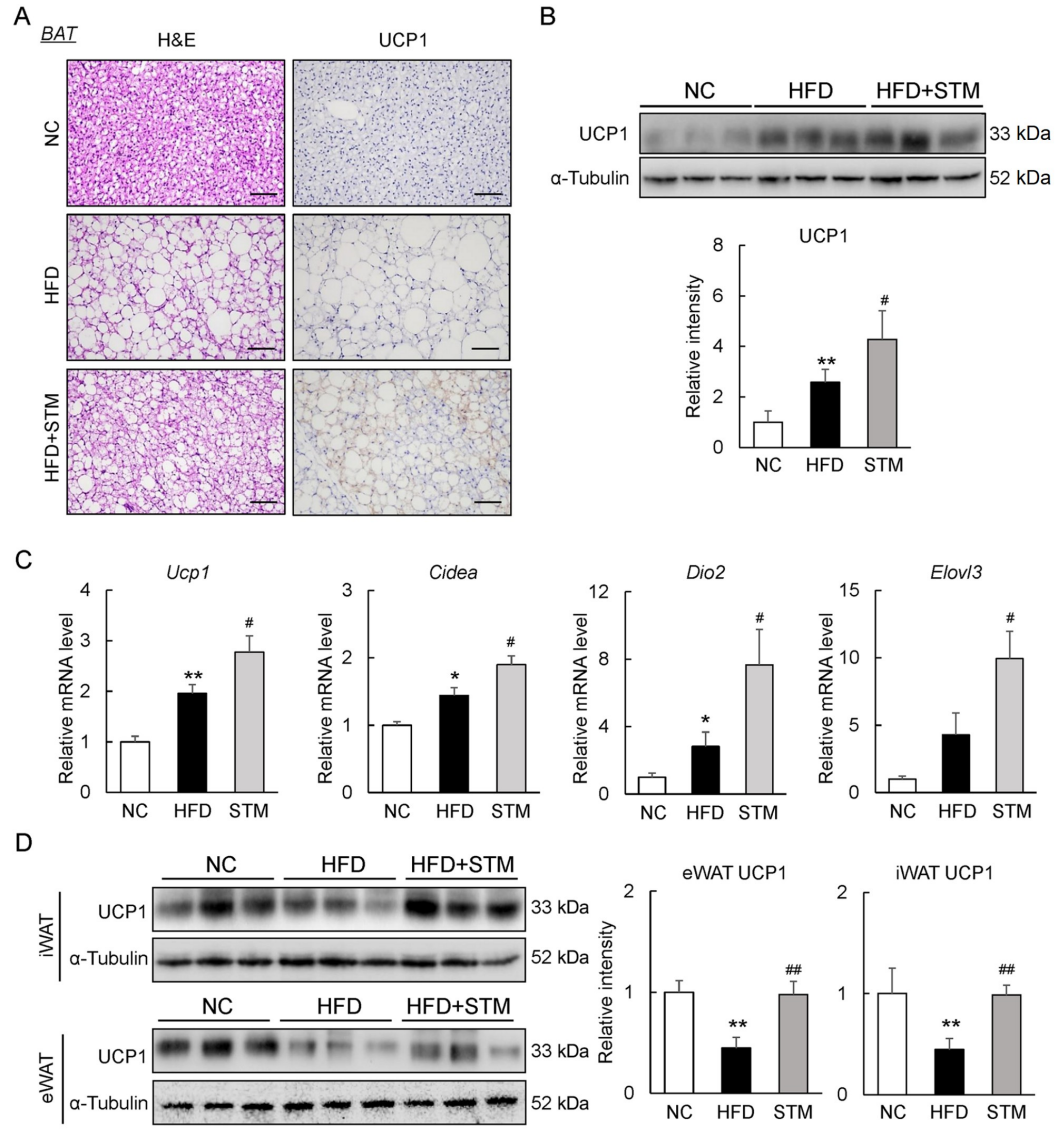
Figure 2 STM supplementation improves BAT function and white adipocyte browning in DIO mice (A) H&E and UCP1 staining of BAT sections (scale bar: 100 μm). (B) Western blot analysis of BAT UCP1. (C) The BAT levels of mRNAs encoding thermogenesis-specific proteins. (D) Western blot analysis of iWAT and eWAT UCP1. Data are presented as the mean±SEM. Significance was determined by one-way ANOVA. * P<0.05, ** P<0.01 vs NC; # P<0.05, ## P<0.01 vs HFD.

BAT engages in nonshivering thermogenesis using fatty acids as substrates and requires activation of mitochondrial UCP1 in brown adipocytes [7]. We found that BAT fat accumulation induced by HFD was reduced by STM ( Figure 2A). The UCP1 mRNA and protein levels were increased by STM ( Figure 2A‒C). The levels of mRNAs encoding thermogenesis-specific proteins, including deiodinase type 2 ( *Dio2*), cell death-inducing DNA fragmentation factor alpha-like effector A ( *Cidea*), and ELOVL fatty acid elongase 3 ( *Elovl3*), were significantly upregulated in STM-treated DIO mice ( Figure 2C). Thus, STM enhanced BAT activity in obese mice.

Beige adipocytes are thermogenic cells that require UCP1 to modulate energy homeostasis [8]. The UCP1 levels in inguinal WAT (iWAT) and epididymal WAT (eWAT) were decreased in HFD-fed mice but were increased notably after STM treatment ( Figure 2D). Thus, STM enhances energy expenditure by promoting BAT activation and fat browning in DIO mice.

### STM increases fatty acid oxidation in skeletal muscles of obese mice

**Figure 3:**
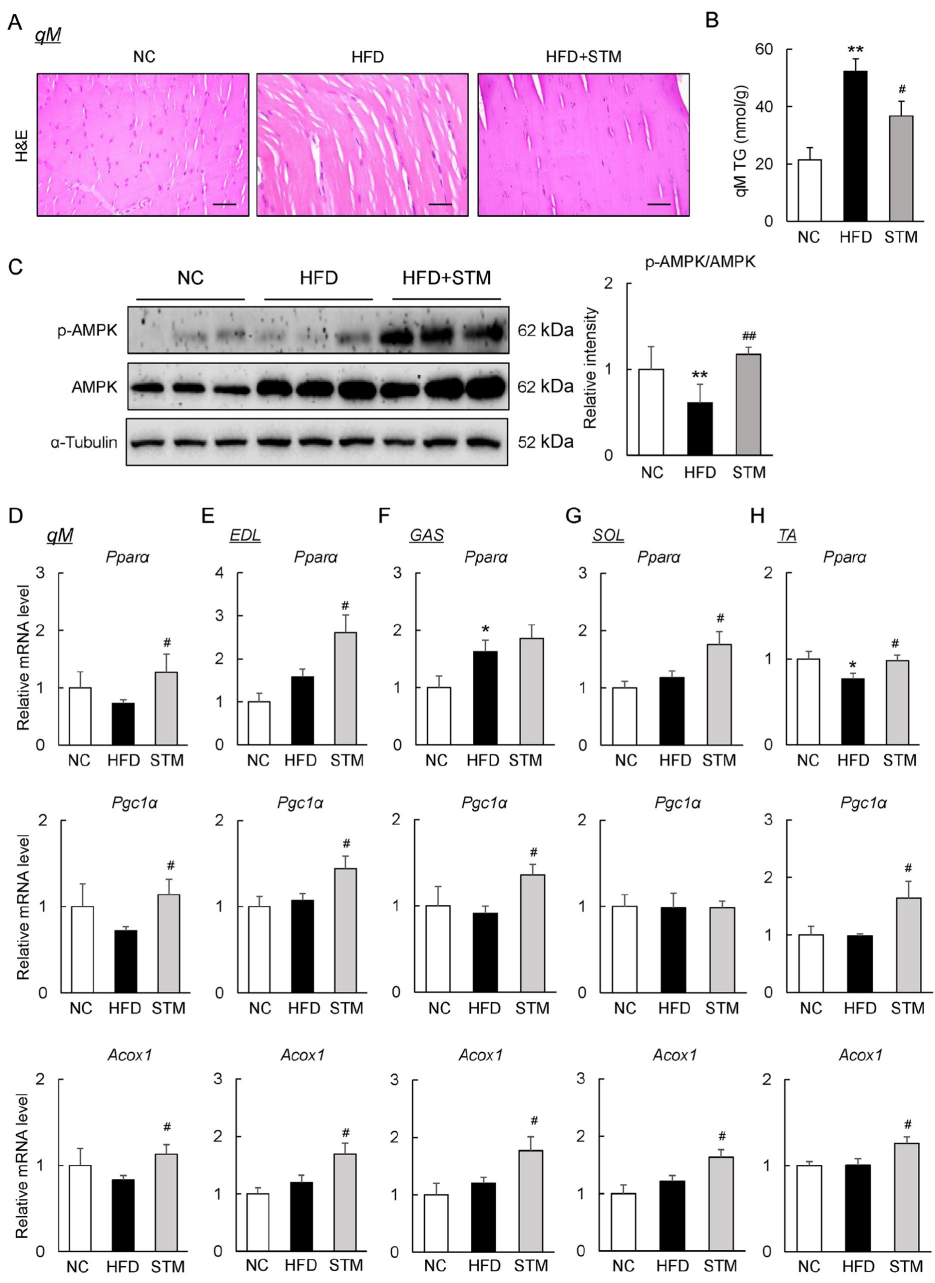
Figure 3 STM supplementation enhances fatty acid oxidation in skeletal muscle (A) Quadriceps muscle (qM) sections stained with H&E (scale bar: 100 μm). (B) qM TG levels. (C) Western blot analysis of qM p-AMPK and AMPK. (D–H) The qM levels of mRNAs encoding lipid oxidation enzymes (D), EDL (E), GAS (F), SOL (G) and TA (H). Data are presented as the mean±SEM. Significance was determined by one-way ANOVA. * P<0.05, ** P<0.01 vs NC; # P<0.05, ## P<0.01 vs HFD.

Skeletal muscle in rodents consumes a great deal of energy produced via fatty acid oxidation [28]. H&E staining revealed that the irregular fiber structure and excess lipid deposition in skeletal muscle (qM) caused by the HFD were restored by STM ( Figure 3A). Furthermore, STM significantly reduced the TG content of the qM of obese mice ( Figure 3B).

To explore the molecular basis of TG accumulation in skeletal muscle, we measured the qM levels of phosphorylated AMPK (a key regulator of energy metabolism). AMPK phosphorylation was decreased in obese mice; STM significantly restored this effect ( Figure 3C). Furthermore, the levels of mRNAs encoding peroxisome proliferator activated receptor alpha (Pparα), peroxisome proliferator-activated receptor-γ coactivator-1α (Pgc1α), and acyl-CoA oxidase (Acox1), all of which play key roles in lipolysis regulation, were significantly increased by STM ( Figure 3D). The relevant genes were also upregulated in other skeletal muscles, including the extensor digitorum longus (EDL), gastrocnemius (GAS), soleus (SOL) and tibialis anterior (TA) muscles, of HFD+STM mice ( Figure 3E–H). Thus, STM improves oxidative metabolism in the skeletal muscles of obese mice.

### STM ameliorates insulin resistance and steatosis in obese mice

**Figure 4:**
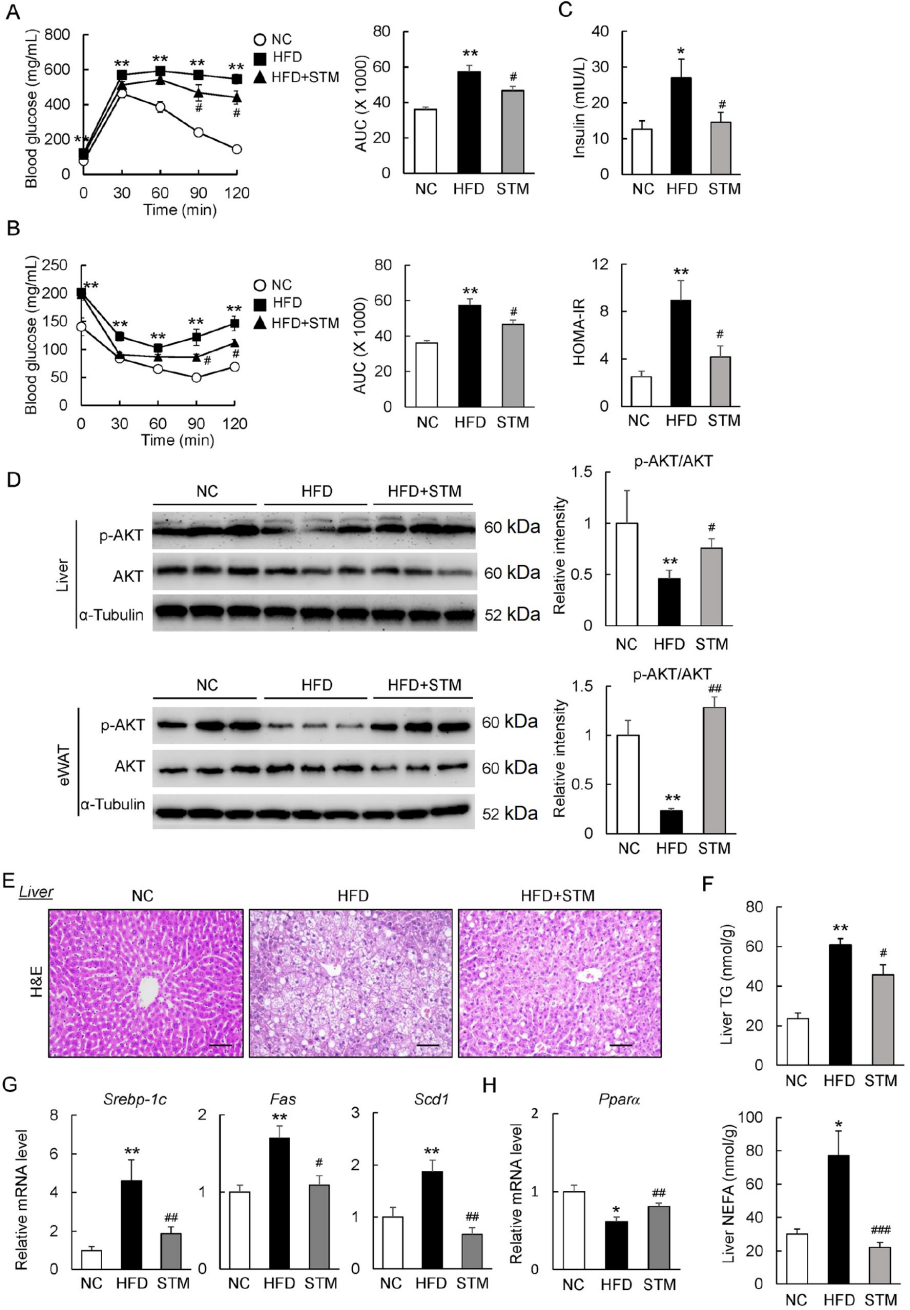
Figure 4 STM supplementation ameliorates HFD-induced insulin resistance and hepatic steatosis (A) GTT and (B) ITT results. (C) Plasma levels of insulin and HOMA-IRs. (D) Western blot analysis of p-AKT and AKT in liver and eWAT. (E) Liver sections stained with H&E (scale bar: 100 μm). (F) Hepatic TG and NEFA levels. (G-H) Liver levels of mRNAs encoding proteins involved in lipid synthesis (G) and lipolysis (H). Data are presented as the mean±SEM. Significance was determined by one-way ANOVA. * P<0.05, ** P<0.01 vs NC; # P<0.05, ## P<0.01 vs HFD.

Next, we determined the therapeutic impact of STM on the abnormal glucose metabolism in HFD-fed mice. The GTT data showed that glucose intolerance was alleviated by STM in DIO mice ( Figure 4A). Insulin resistance was also improved in HFD+STM mice (as assessed by the ITT) ( Figure 4B). Indeed, the high insulin level in HFD mice was decreased by STM ( Figure 4C), and HOMA-IR was also suppressed ( Figure 4C). The HFD-induced decrease in the phosphorylation of protein kinase B (AKT), a downstream key insulin-signal kinase, was restored in the liver and fat of HFD+STM mice ( Figure 4D).

HFD-induced hepatic lipid accumulation was attenuated in STM-treated mice ( Figure 4E). The liver TG and NEFA contents in the DIO mice were reduced by STM ( Figure 4F), indicating that STM improved hepatic steatosis. Furthermore, the expressions of genes encoding sterol regulatory element-binding protein 1C (Srebp-1c), fatty acid synthase (Fas), and stearoyl-CoA desaturase 1 (Scd1) (key genes of *de novo* lipogenesis) were significantly inhibited by STM ( Figure 4G). Conversely, the expression of the lipolytic gene *Pparα* was increased by STM ( Figure 4H). Together, the data suggest that STM could be used to treat obesity-related insulin resistance and NAFLD.

### STM suppresses inflammation in the adipose tissue and liver of obese mice

**Figure 5:**
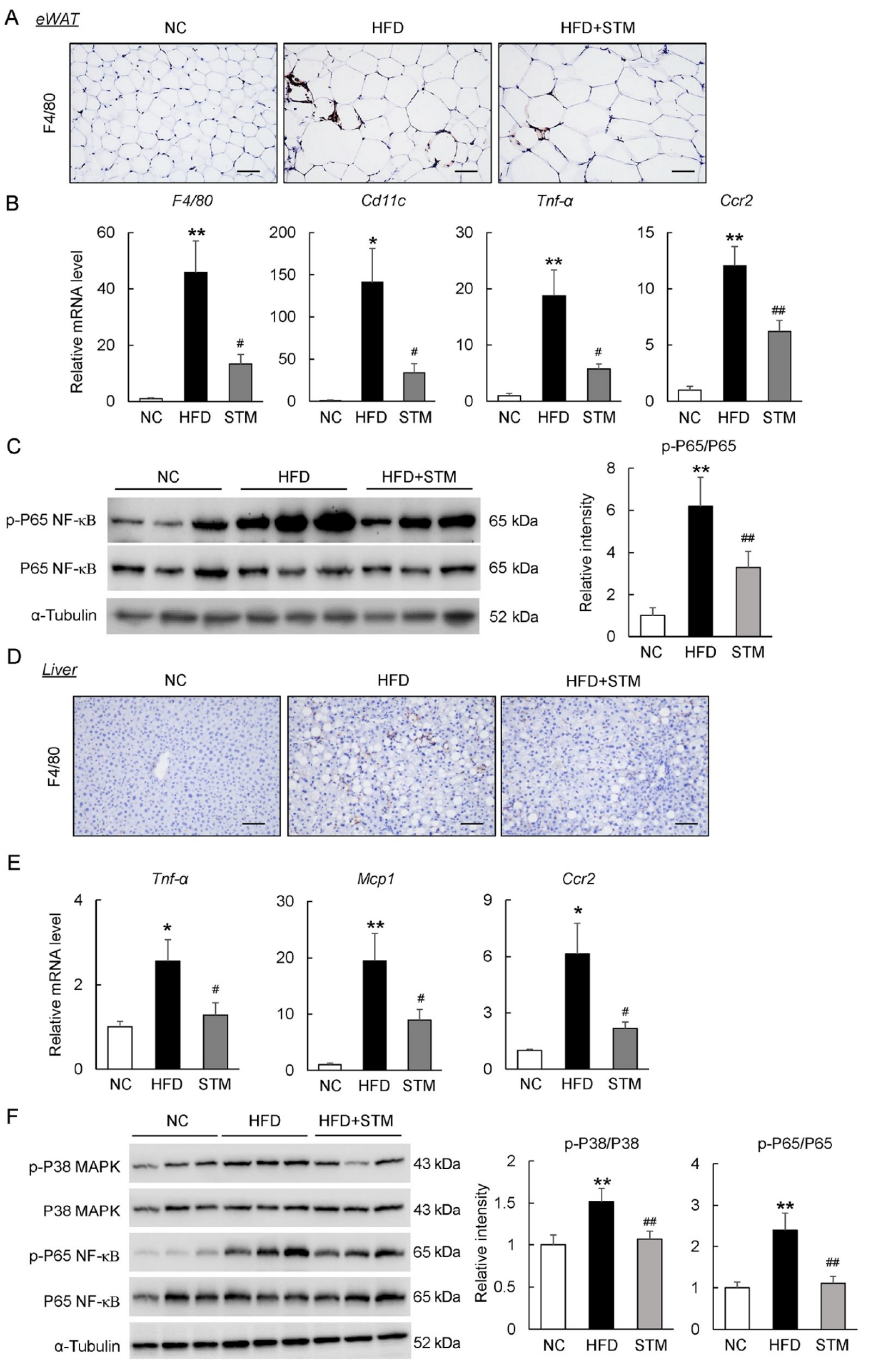
Figure 5 STM supplementation suppresses fat and liver inflammation in obese mice (A) F4/80 immunostaining of eWAT sections (scale bar: 100 μm). (B) eWAT levels of mRNAs encoding proinflammatory proteins. (C) Western blot analysis of p-NF-κB and NF-κB in eWAT. (D) F4/80 immunostaining of liver sections (scale bar: 100 μm). (E) Liver levels of mRNAs encoding proinflammatory proteins. (F) Western blot analysis of liver p-NF-κB and p-P38 MAPK and the total protein contents. Data are presented as the mean±SEM. Significance was determined by one-way ANOVA. * P<0.05, ** P<0.01 vs NC; # P<0.05, ## P<0.01 vs HFD.

Next, we examined the therapeutic effect of STM on HFD-related inflammation in metabolic tissues. As shown in Figure 5A, mice treated with STM exhibited less macrophage infiltration (as evidenced by staining for the F4/80 macrophage marker) than mice fed with a HFD. STM suppressed the expression levels of mRNAs encoding F4/80 and proinflammatory cytokines, including cluster of differentiation 11c (CD11c), tumor necrosis factor-α (TNF-α), and C-C motif chemokine receptor 2 (Ccr2), in the eWAT of HFD-fed mice ( Figure 5B). Such changes were associated with reduced phosphorylation of nuclear factor-κB (NF-κB) p65 in eWAT ( Figure 5C). Additionally, STM significantly reduced the number of F4/80 ^+^ cells in the liver, the expressions of proinflammatory markers (TNF-α, Ccl2 and Ccr2), and the activation of NF-κB and p38 mitogen-activated protein kinase (MAPK) signaling ( Figure 5D–F). Therefore, STM reversed HFD-induced chronic inflammation.

### STM reversal of obesity does not depend on gut microbiota improvement

**Figure 6:**
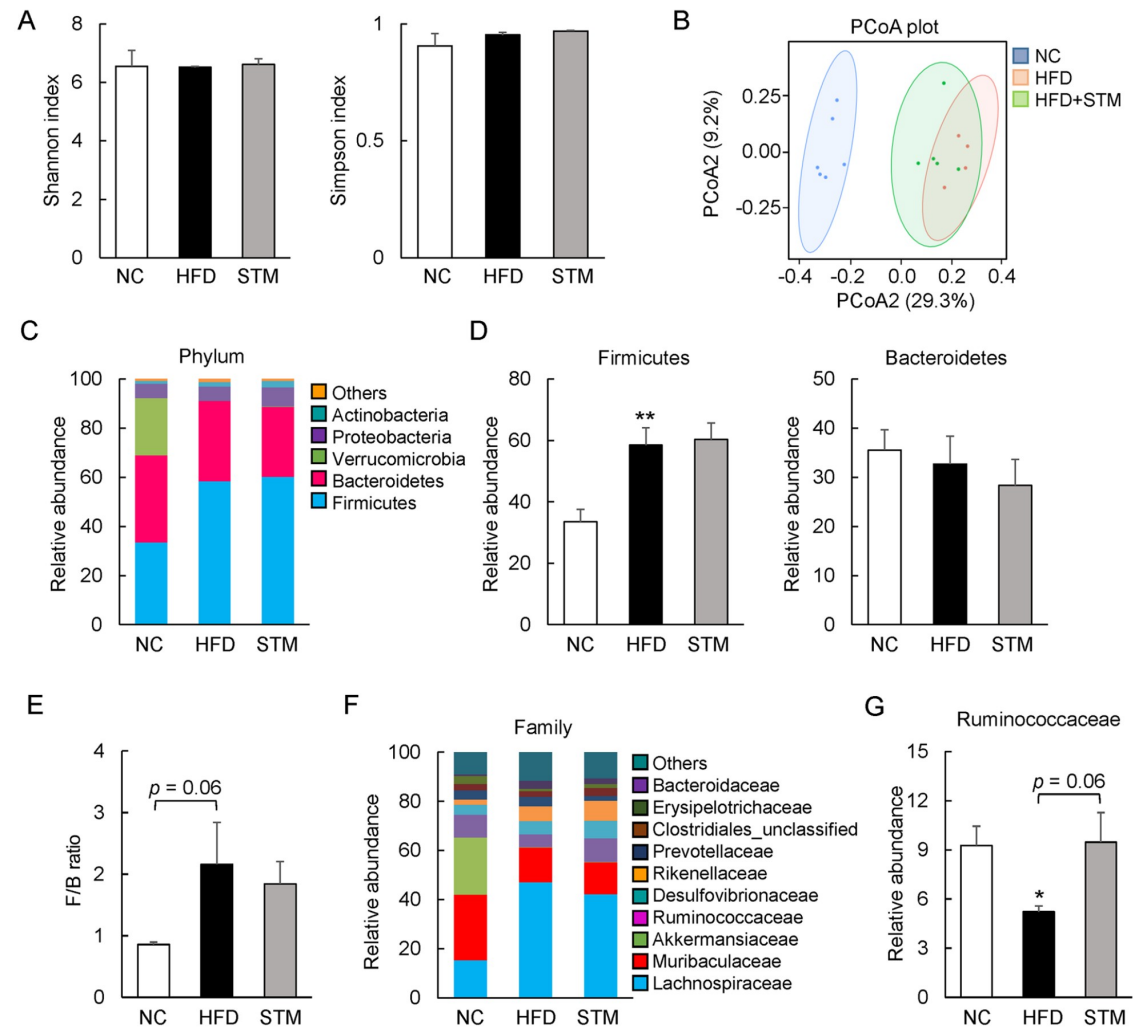
Figure 6 STM supplementation fails to alleviate dysbiosis of the gut microbiota (A) The alpha diversity of the gut microbiota was evaluated by deriving the Shannon (left) and Simpson (right) indices. (B) PCoA cluster plots of beta diversity reveal the among-population distances of each group. (C) Relative abundances of bacterial phyla. (D) Relative abundances of Firmicutes (left) and Bacteroidetes (right). (E) The Firmicutes-to-Bacteroidetes ratios. (F) Relative abundances of bacterial families. (G) Relative abundances of Ruminococcaceae. Data are presented as the mean±SEM. Significance was determined by one-way ANOVA. * P<0.05, ** P<0.01 vs NC; # P<0.05, ## P<0.01 vs HFD.

Gut microbiotic dysbiosis is strongly associated with obesity progression [29]. However, it is not known whether STM regulates the gut microbiome in HFD-fed mice. We sequenced the 16S rRNA genes of the intestinal contents. The Shannon and Simpson alpha-diversity indices did not significantly differ among the groups ( Figure 6A). To explore beta diversity, we used principal coordinate analysis (PCoA) employing the weighted UniFrac distance metric ( Figure 6B). NC and HFD mice displayed distinct clustering of their microbial community structures ( *P<*0.001); the HFD+STM group structure was similar to that of HFD mice ( Figure 6B). In terms of the predominant microbial phyla, the level of Firmicutes was increased in HFD mice, but the level of Bacteroidetes did not differ between HFD and NC mice, thus increasing the ratio of Firmicutes to Bacteroidetes ( Figure 6C–E). Unexpectedly, STM did not affect the composition or proportions of Firmicutes and Bacteroidetes in obese mice ( Figure 6C–E). Furthermore, the levels of intestinal bacteria at the family level were comparable between HFD and HFD+STM mice, although that of Ruminococcaceae, an intestinal probiotic, was slightly increased by STM ( Figure 6F,G). These results suggest that STM does not reprogram the gut microbiota.

## Discussion

Mechanistically, obesity is caused by excess food intake and/or insufficient calorie utilization, resulting in insulin resistance and inflammation. Therefore, strategies to combat obesity and its associated metabolic abnormalities include attenuation of calorie intake, increased energy utilization, and regulation of lipid metabolism. Indeed, obesity-related morbidity has increased remarkably over the past decades [2]. Thus, a therapeutic agent protecting against obesity is urgently needed.

We previously showed that STM has a preventative effect on diet-induced obesity. STM dose-dependently attenuated HFD-associated weight gain, insulin resistance, and inflammation [20]. However, the potential mechanisms need to be explored. More importantly, compared to a preventative study, a therapeutic experiment is more relevant to the human situation; STM reduces obesity. We found that STM at 0.01% (w/w) (the low dose of the preventative study) failed to modulate energy homeostasis in obese mice (data not shown). However, STM at 0.1% (w/w) strikingly reversed obesity progression and significantly reduced the associated inflammation and insulin resistance. Unexpectedly, we found that STM increased energy expenditure and fat browning and enhanced fatty acid oxidation in adipose tissues and skeletal muscle. Together, the data show that STM protects against obesity by promoting oxidative metabolism in mice with preexisting obesity.

STM is a bitter secoiridoid glycoside that might affect food intake in rodents if mixed with chow. Therefore, we administered STM by gavage to avoid any influence on calorie intake. STM markedly decreased the weight gain of obese mice, not because food intake was reduced (data not shown) but because energy expenditure was increased. STM increases fatty acid β-oxidation in adipose tissue by upregulating *Kat-1*, which encodes 3-ketoacyl-coA-thiolase, a critical enzyme of fatty acid β-oxidation, preventing HFD-induced hyperlipidemia and insulin resistance [18]. STM in the present study increased oxygen consumption of obese mice, suggesting enhancement of fat oxidation. In addition, it promoted UCP1 expression in both classic BAT and beige WAT, increasing TG combustion and energy expenditure. Although the mechanisms by which STM regulates brown fat function and browning remain obscure, we can conclude that administration of STM restores obesity partially by regulating energy homeostasis in DIO mice. Of course, how STM activates the UCP1 pathway in adipose tissues warrants further investigation.

AMPK is a major nutrient sensor and plays a critical role in modulating energy balance modulation in metabolic tissues, such as skeletal muscle and liver. AMPK activation suppresses lipogenesis and increases fatty acid oxidation via phosphorylation of ACC [30]. A recent study revealed that STM stimulated AMPK production in HepG2 cells and mitigated hepatocellular fat accumulation [19]. In this study, STM enhanced the phosphorylation of skeletal muscle AMPKα and increased the levels of mRNAs encoding proteins involved in fatty acid β-oxidation. Thus, STM causes weight reduction by increased energy expenditure and lipolysis.

Several studies have indicated that STM improves obesity-related metabolic disorders, including diabetes [ 31– 33] and NAFLD [ 16, 34] . Obesity activates the NF-κB pathway and increases the expressions of proinflammatory cytokines such as TNF-α and interleukin-6 (IL-6) in adipose tissue, thereby disrupting insulin signaling and triggering insulin resistance and NAFLD [35]. STM significantly reduces NF-κB pathway-mediated activation of proinflammatory cytokine release in animals and LPS-induced RAW 264.7 macrophages [36]. *In vitro*, it enhances the synthesis of important insulin-signaling-associated proteins, including phosphorylated insulin receptor and Akt [19]. The GTT and ITT data in the present study revealed that STM treatment significantly promoted obesity-associated glucose tolerance and insulin sensitivity, although overnight fasting may not be optimal in our GTT test and a short time (4‒6 h) fasting may be more appropriate [37]. Furthermore, STM inhibited obesity-induced inflammation by decreasing macrophage infiltration and proinflammatory signaling and restored insulin sensitivity to the WAT and liver. Therefore, STM may suppress both insulin resistance and hepatic steatosis.

More and more preclinical and clinical evidence highlights the critical roles played by the intestinal microbiota in obesity and its associated metabolic diseases [38]. The Firmicutes-to-Bacteroidetes ratio is correlated positively with obesity progression. Thus, novel therapies that regulate intestinal bacteria might prevent or treat obesity. As expected, HFD induced gut microbiotic dysbiosis, thereby increasing the Firmicutes level and the Firmicutes-to-Bacteroidetes ratio. However, STM did not affect the intestinal microbiotic composition at the phylum or family levels and did not reduce the Firmicutes/Bacteroidetes numbers. Thus, STM-induced reversal of dietary obesity does not involve changes in the intestinal microecology.

In conclusion, STM reduces diet-induced obesity by regulating energy homeostasis and enhancing oxidative metabolism, and mitigates the associated insulin resistance and NAFLD. Thus, STM may serve as a potential pharmacological strategy for the treatment of obesity.
